# The Correlation between the Severity of Obstructive Sleep Apnea and Insulin Resistance in a Japanese Population

**DOI:** 10.3390/jcm13113135

**Published:** 2024-05-27

**Authors:** Yukako Tomo, Ryo Naito, Yasuhiro Tomita, Satoshi Kasagi, Tatsuya Sato, Takatoshi Kasai

**Affiliations:** 1 Sleep Center, Toranomon Hospital, Tokyo 105-0001, Japan; yukazou@gmail.com (Y.T.); ytomitatmy@gmail.com (Y.T.); skasagi@knd.biglobe.ne.jp (S.K.); kasai-t@mx6.nisiq.net (T.K.); 2 Department of Cardiovascular Biology and Medicine, Juntendo University Graduate School of Medicine, Tokyo 113-8421, Japan; 3 Cardiovascular Respiratory Sleep Medicine, Juntendo University Graduate School of Medicine, Tokyo 113-8421, Japan; 4 Department of Cellular Physiology and Signal Transduction, Sapporo Medical University School of Medicine, Sapporo 060-8556, Japan; sato.tatsuya@sapmed.ac.jp; 5 Department of Cardiovascular, Renal and Metabolic Medicine, Sapporo Medical University School of Medicine, Sapporo 060-8556, Japan

**Keywords:** obstructive sleep apnea, insulin resistance, HOMA-IR

## Abstract

**Background:** Repetitive episodes of apnea and hypopnea during sleep in patients with obstructive sleep apnea (OSA) are known to increase the risk of atherosclerosis. Underlying obesity and related disorders, such as insulin resistance, are indirectly related to the development of atherosclerosis. In addition, OSA is independently associated with insulin resistance; however, data regarding this relationship are scarce in Japanese populations. **Methods:** This study aimed to examine the relationship between the severity of OSA and insulin resistance in a Japanese population. We analyzed the data of consecutive patients who were referred for polysomnography under clinical suspicion of developing OSA and who did not have diabetes mellitus or any cardiovascular disease. Multiple regression analyses were performed to determine the relationship between the severity of OSA and insulin resistance. **Results:** The data from a total of 483 consecutive patients were analyzed. The median apnea-hypopnea index (AHI) was 40.9/h (interquartile range: 26.5, 59.1) and the median homeostasis model assessment for insulin resistance (HOMA-IR) was 2.00 (interquartile range: 1.25, 3.50). Multiple regression analyses revealed that the AHI, the lowest oxyhemoglobin saturation (SO_2_), and the percentage of time spent on SO_2_ < 90% were independently correlated with HOMA-IR (an adjusted R-squared value of 0.01278821, *p* = 0.014; an adjusted R-squared value of −0.01481952, *p* = 0.009; and an adjusted R-squared value of 0.018456581, *p* = 0.003, respectively). **Conclusions:** The severity of OSA is associated with insulin resistance assessed by HOMA-IR in a Japanese population.

## 1. Introduction

Cardiovascular disease (CVD) is a leading cause of death worldwide. Knowing how to prevent CVD has been key for promoting health of the population and individuals around the world. The American Heart Association created a definition for the construct of cardiovascular health (CVH) in 2010 based on the idea that health is not regarded as merely the absence of disease [1]. It leveraged relevant existing evidence and emerging prevention concepts to formulate a definition that was intended to be accessible for all stakeholders, such as individuals, health practitioners, researchers for health, and policymakers, in order to focus efforts on improving CVH for all individuals. The initial definition of CVH was based on seven health behaviors, including indicators of dietary quality; participation in physical activity; exposure to cigarette smoking; and measures of body mass index, fasting blood glucose, total cholesterol, and blood pressure levels. Recently, the CVH has been updated with the inclusion of sleep as a novel CVH component [1]. Sleep is fundamental for human biology and essential for life. Epidemiological studies have identified inappropriate sleep hygiene as a risk factor for all-cause mortality, and subsequent research has explored potential mechanisms, including implications for cardiometabolic health. Much of the existing research has focused on sleep duration; however, sleep health is a multidimensional construct with overlapping components, such as duration, regularity, efficiency, self-satisfaction, impact on daytime alertness, as well as sleep-disordered breathing (SDB) [1].

Obstructive sleep apnea (OSA), which is a main type of SDB, is associated with the incidence and progression of metabolic and atherosclerotic diseases, including coronary artery disease, hypertension, and diabetes mellitus (DM) [2,3,4,5]. DM is mainly driven by insulin resistance and impaired secretion. Epidemiological studies have reported that OSA is associated with insulin resistance independent of confounders, such as obesity [6,7]. In a study of 150 overweight men, the apnea-hypopnea index (AHI) was associated with insulin resistance, independent of obesity [6]. Another case–control study of non-obese young men reported an association between OSA and insulin resistance, suggesting that OSA may provoke insulin resistance independent of obesity and age [7]. Japanese patients with OSA have different characteristics, such as comorbid obesity and anatomical abnormalities of the upper airway, from those in Europe and the United States. However, it remains unconclusive whether the severity of OSA in patients with OSA in Japan is associated with insulin resistance, even in the absence of diabetes mellitus and CVDs, which leads to insulin resistance with increased pro-inflammatory status. Therefore, we aimed to examine the relationship between the severity of OSA and insulin resistance in a Japanese population without diabetes mellitus and CVDs through exploratory data analyses.

## 2. Materials and Methods

### 2.1. Study Population

This is a retrospective observational study conducted at a single institution. Consecutive patients diagnosed with OSA using polysomnography at the sleep center of Toranomon Hospital, Tokyo, Japan, between 1 January 2006 and 1 October 2006, were enrolled in the study.

The exclusion criteria were as follows: (1) the presence of DM or undertreatment with any antidiabetic medication; (2) the presence of any CVD, including coronary artery disease, heart failure, or stroke; and (3) a history of renal failure undergoing dialysis treatment. 

This study was conducted in accordance with the Declaration of Helsinki and was approved by the Ethics Board of Toranomon Hospital. In this study, sleep studies, anthropometric data collection, and blood sampling, which had already been performed as a routine clinical checkup, were analyzed. The requirement to obtain informed consent was waived by the Toranomon Hospital Ethics Board using opt-out methods.

### 2.2. Sleep Study

For sleep studies, overnight polysomnography was carried out, according to the standard protocols and criteria [8]. Electrocardiography, electroencephalography, electrooculography, and electromyography were performed, and thoracoabdominal motion was monitored with respiratory inductance plethysmography. Airflow was measured with an oronasal thermal airflow sensor and nasal pressure cannula, and oxyhemoglobin saturation (SO_2_) was monitored with oximetry. Respiratory events (apneas or hypopneas) were counted according to the American Academy of Sleep Medicine scoring manual 2020 updates [9]. Apnea with and without rib cage and/or abdominal movement were defined as obstructive and central apnea, respectively. Hypopnea was defined as obstructive if any of the following conditions were present: (1) paradoxical chest or abdominal movements, (2) snoring, or (3) flow limitation during hypopnea events. Otherwise, hypopnea was classified as central.

### 2.3. Index of Insulin Resistance

The homeostasis model assessment for insulin resistance (HOMA-IR) was used as an index of insulin resistance, which was calculated as the fasting serum insulin level multiplied by the fasting glucose level multiplied by 405 [10]. Serum insulin and fasting glucose levels were measured using a commercially available assay at Toranomon Hospital before the polysomnography was performed.

### 2.4. Other Variables

The following variables were obtained from the clinical chart at the time of polysomnography: age; sex; body mass index (BMI); waist circumference; the presence of hypertension; and serum levels (total cholesterol, triglycerides, low-density lipoprotein cholesterol, high-density lipoprotein cholesterol, insulin, uric acid, and C-reactive protein). BMI was calculated as the body weight in kilograms divided by the square of body height in meters, and waist circumference was measured around the abdomen at the level between the top of the hip bone and the bottom of the ribs at the time of polysomnography. Hypertension is defined as systolic blood pressure ≥ 140 mm Hg, diastolic blood pressure ≥ 90 mm Hg, or under any antihypertensive medications.

### 2.5. Outcomes

Relationships between OSA severity, AHI, 3% oxygen desaturation index (ODI), lowest SO_2_, the percentage of time spent on SO_2_ < 90% (%TST SO_2_ < 90%), arousal index, and HOMA-IR were examined.

### 2.6. Statistical Analysis

The clinical data were presented as the mean ± standard deviation or median and interquartile range. Correlation analyses were performed to evaluate relationships between each index of OSA severity (AHI, 3% ODI, lowest SO_2_, %TST SO_2_ < 90%, and arousal index) and HOMA-IR as the dependent variable adjusted for covariates (age, sex, and BMI), and the coefficient, standard error, t-test statistic (T), adjusted R-squared value, and the *p*-value were calculated. The indices of OSA severity were AHI, 3% ODI, lowest SO_2_, %TST SO_2_ < 90%, and arousal index. As HOMA-IR was not normally distributed, log-transformed HOMA-IR (log HOMA-IR) was used in the analyses. Multiple regression analyses were performed to determine the association between OSA severity and HOMA-IR, with the other variables obtained at the time of polysomnography. Statistical significance was set at *p* < 0.05. All statistical analyses were performed using the SPSS statistical software (version 11.0; SPSS Inc., Chicago, IL, USA).

## 3. Results

A total of 483 patients were enrolled in this study. The participants’ characteristics are presented in Table 1. The median age was 55.0 years (interquartile range (IQR): 44, 64) and 90.9% of the study population were men. The prevalence of hypertension was 54.0% and the median HOMA-IR was 2.00 (IQR: 1.25, 3.50). The polysomnographic data are presented in Table 2. The median AHI was 40.9 (IQR: 26.5, 59.1), the 3% ODI was 28.4 (IQR: 13.3, 50.5), the lowest SO_2_ was 77 (IQR: 69, 82), the median arousal index was 39.1 (26.6, 56.3), and the median %TST SO_2_ < 90% was 14.6 (IQR: 3.8, 44.3). 

The relationships between the polysomnographic data and log HOMA-IR are shown in Table 3. The AHI, 3% ODI, %TST SO_2_ < 90%, and arousal index were positively correlated with log HOMA-IR (an adjusted R-squared value of 0.0038321, *p* = 0.015 for AHI; an adjusted R-squared value of 0.0030430, *p* = 0.024 for 3% ODI; an adjusted R-squared value of 0.003599, *p* = 0.001 for %TST SO_2_ < 90%; and an adjusted R-squared value of 0.00343508, *p* = 0.028 for the arousal index). The lowest SO_2_ was inversely correlated with log HOMA-IR (an adjusted R-squared value of −0.0061958, *p* = 0.004). Multiple regression analyses revealed that AHI, 3% ODI, lowest SO_2_, and %TST SO_2_ < 90% were independently correlated with log HOMA-IR (an adjusted R-squared value of 0.01278821, *p* = 0.014 for AHI; an adjusted R-squared value of −0.01481952, *p* = 0.009 for lowest SO_2_; and an adjusted R-squared value of 0.018456581, *p* = 0.003 for %TST SO_2_ < 90%) (Table 4).

## 4. Discussion

Our study demonstrated that the indices of OSA severity (AHI, lowest SO_2_, and %TST SO_2_ < 90%) were correlated with insulin resistance, as assessed by HOMA-IR after adjusting for covariates in a Japanese population without diabetes mellitus and CVDs. The finding seems to be pathophysiologically valid because the high severity of OSA can be related to chronic sympathetic activity and systemic inflammation that can elicit insulin resistance. Although sleep disorders are often readily missed in routine medical care, they have been reported to be strongly associated with the development of CVDs. Therefore, the results of this study suggest that, at least in Japanese populations, even in the absence of known CVDs or diabetes mellitus, findings of insulin resistance may form the basis for suspecting the presence of OSA. 

Epidemiological studies have reported an association between SDB and insulin resistance. A study reported that increased AHI was associated with worsening insulin resistance (odds ratio, 2.15; 95% confidence interval, 1.05 to 4.38), independent of obesity, in 150 obese men (mean BMI 30.5 ± 2.9 kg/m^2^) without DM or cardiopulmonary disease [6]. Another case–control study of 52 young lean men (with a mean age of 23.4 ± 0.4 years and mean BMI of 22.6 ± 0.3 kg/m^2^) without cardiometabolic disease reported that participants with OSA had 27% lower insulin sensitivity, estimated by the Matsuda index, and 37% higher insulin secretion after the ingestion of glucose load than those without OSA [7]. A longitudinal study assessing 141 non-diabetic men (with a mean age of 57.5 years and a mean BMI of 26.9 kg/m^2^), with a mean follow-up of 11 years and 4 months, reported that an oxygen desaturation index >5/h was significantly associated with deteriorated insulin resistance assessed using the change in HOMA-IR from baseline to follow-up and a higher incidence of diabetes, partially supporting the findings of our study [11]. Nevertheless, the strong correlation between the severity of OSA observed in this study with insulin resistance reemphasizes that the presence of OSA can be a potent risk factor for atherosclerotic diseases via increased insulin resistance.

### 4.1. Mechanisms of the Association between OSA and Insulin Resistance

**Figure 1:**
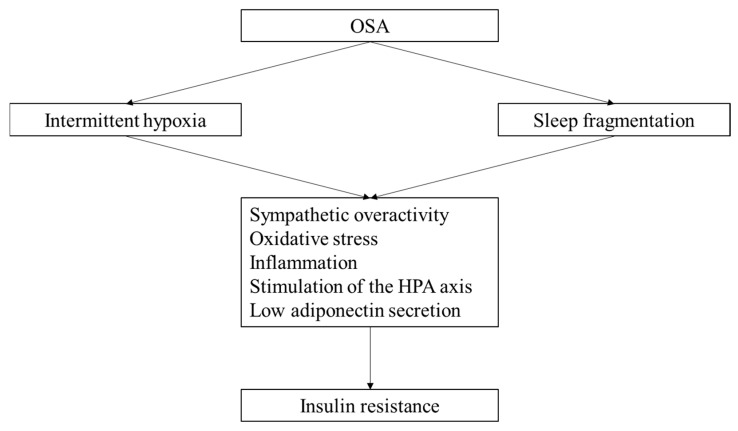
Mechanisms linking OSA and insulin resistance include sympathetic overactivity, oxidative stress, inflammation, the stimulation of the HPA axis and low adiponectin secretion induced by intermittent hypoxia and sleep fragmentation. HPA; hypothalamic–pituitary–adrenal.

The presumed mechanisms underlying the association between OSA and insulin resistance include intermittent hypoxemia caused by OSA [12], inappropriate OSA-related sleep hygiene [13,14,15], increased sympathetic nerve activity, oxidative stress, and inflammation (Figure 1). In experimental animal models, intermittent hypoxia was shown to mediate hypoxia-inducible factor 1α expression in pancreatic beta cells, resulting in insulin resistance when the production of reactive oxygen species increases [16]. It has been speculated that initial hypoxia exposure may affect insulin clearance in the liver. From these findings, it is possible that the effects of hypoxia on insulin resistance may differ depending on the degree of hypoxia and the duration of exposure. Sleep fragmentation using auditory and mechanical stimulation in healthy subjects has been reported to reduce insulin sensitivity by 20–25% [15,17,18]. The mechanisms by which SDB induces insulin resistance is likely to be complex and cannot be explained by a single pathway, but the pathogenesis listed above may at least be involved in the sleep disturbances that can develop or exacerbate CVDs via the presence of insulin resistance.

### 4.2. Positive Airway Pressure Therapy for OSA and Insulin Resistance

Treatments for OSA include lifestyle modification mainly for obesity, postural therapy for patients with OSA whose severity of OSA fluctuate according to the body position, intraoral appliance, upper airway surgery, and CPAP therapy. Among the various treatment options for OSA, CPAP therapy has been an established choice to decrease AHI and improve symptoms due to OSA and quality of life, which has been covered by insurance since 1998 in Japan [2]. Variety of beneficial effects of CPAP for patients with OSA have been reported such as lowering blood pressure, suppression of sympathetic nerve activity, decrease in inflammatory markers, improvement of vascular endothelial function, left ventricular systolic and diastolic function, and nocturnal myocardial ischemia [2]. However, prognostic benefits of CPAP in patients with OSA concomitant with CVDs have been conflicting. An observational study examining prognostic effects of CPAP therapy in fifty-four patients with OSA and coronary artery disease reported that CPAP therapy was associated with reduction in a composite of cardiovascular events (cardiovascular death, acute coronary syndrome, hospitalization for heart failure, or coronary revascularization) with hazard ratio of 0.24 (95% confidence interval of 0.09–0.62) for CPAP as compared to the non-treatment group during a median follow-up of 86.5 months [19]. In contrast, large-scale randomized controlled trials investigating the effects of CPAP in patients with OSA and coronary artery disease did not agree with this finding, although low adherence to CPAP (with a mean usage time of CPAP < 4 h/night) was found to potentially affect the observed neutral finding [20,21,22]. The most recent trial of 1264 patients with acute coronary syndrome and OSA compared a range of cardiovascular events, including cardiovascular death, non-fatal myocardial infarction, non-fatal stroke, hospitalization for unstable angina pectoris, heart failure, and transient ischemic attack between CPAP and non-CPAP groups [22]. No significant reduction in the incident cardiovascular events was observed for the CPAP group (with a hazard ratio of 0.89 and a 95% confidence interval of 0.68–1.17) during a median follow-up of 3.35 years. Similarly, the prognostic benefits of CPAP therapy on reducing the number of cardiovascular events in patients with CVDs, including heart failure, ventricular arrhythmias, stroke, aortic disease, and other vascular diseases, have not yet been demonstrated in randomized controlled trials. 

Although our study demonstrated the relationship between the severity of OSA and insulin resistance, evidence on whether positive airway pressure therapy for OSA improves insulin resistance has not yet been established. A small randomized controlled trial that assessed the effect of CPAP therapy on glycated hemoglobin in 50 patients with OSA and type 2 DM (with a mean age of 61 ± 9 years and a mean BMI of 32.5 ± 4.5 kg/m^2^) reported that CPAP therapy for 6 months decreased glycated hemoglobin compared to the no-CPAP group (with an intergroup adjusted difference of −0.4 (95% confidence interval of −0.7 to −0.04), *p* = 0.029) [23]. HOMA-IR was also significantly reduced in the CPAP group (with an intergroup adjusted difference of −2.58 and a 95% confidence interval of −4.75 to −0.41, *p* = 0.023). Furthermore, serum biomarker levels of IL-1β, IL-6, and adiponectin also improved in the CPAP group compared with the control group, suggesting the effects of CPAP therapy on improving glucose metabolism and reversing proinflammatory status [23]. Another randomized trial assessed the incremental effect of combined intervention, including a weight loss intervention and CPAP, in which 146 patients with obesity, moderate-to-severe OSA, and serum levels of C-reactive protein (CRP) greater than 1.0 mg/L were allocated to three groups (CPAP alone, weight loss intervention alone, or CPAP with a weight loss intervention) [24]. In the 24th week of the interventions, CRP levels, insulin resistance levels, and serum triglyceride levels were reduced in the patients assigned to weight loss only and those assigned to the combined interventions, while none of these changes were observed in the group treated using CPAP alone. Reductions in insulin resistance and serum triglyceride levels were greater in the combination treatment group than in the group treated using CPAP therapy alone. In per-protocol analyses, which included 90 participants who met prespecified criteria for adherence to CPAP therapy (used for an average of at least 4 h per night on at least 70% of the total number of nights) [24]. On the other hand, a study in which the effects of CPAP on glycemic variability were assessed in 203 patients of SDB with or without diabetes mellitus (mean age 67.5 ± 14.1 years) was also conducted. Glycemic variability assessed by continuous glucose monitoring showed that CPAP reduced the mean amplitude of glycemic excursion from 75.3 to 53.0 mg/dl in the non-DM group, but a similar finding was not observed in the DM group, suggesting the difficulty of improving glucose metabolism using CPAP therapy in patients with advanced glucose metabolism disorders [25]. A meta-analysis of nine randomized controlled trials (443 patients) comparing CPAP treatment with sham CPAP groups, placebo groups, or no-treatment groups, with the goal of improving insulin resistance and glucose metabolism in non-diabetic adults with OSA, reported that CPAP therapy significantly improved HOMA-IR (with a mean difference = −0.39 Ui (CI: −0.69 to −0.08), *p* < 0.05, and I^2^ = 57%) as compared to the non-CPAP group, while no significant differences in fasting glucose was observed [26]. The other meta-analysis, in which 23 studies (19 prospective studies and 4 randomized controlled trials with a total of 965 patients) were included, assessed the effect of CPAP therapy on HOMA-IR, fasting blood glucose, and fasting insulin in non-diabetic and pre-diabetic patients with OSA. CPAP therapy showed significant reductions in the pooled standard difference regarding the means of HOMA-IR (−0.442, *p* = 0.001) from baseline levels compared with the control group, while no significant differences were observed for fasting blood glucose and fasting insulin from baseline levels between the CPAP and the control groups [27]. Despite the findings of these meta-analyses, we cannot conclude the generalizability of these findings due to the small sample size of each study included in the meta-analyses and the difference in study populations and designs. Further large-scale studies are needed to determine the effects of CPAP on insulin resistance. Given that diets play an important role in the development of obesity which can cause OSA and cardiovascular disease through metabolic disorders and inflammation, and given that specific diets such as Washoku (Japanese diet) and the Mediterranean diet have been regarded as healthy diets [28], research on dietary patterns in relation to the prevention of cardio-metabolic diseases is warranted in order to reduce the burden of CVDs. Furthermore, the development of artificial intelligence may enable the implementation of personalized medicine in various medical fields, such as sleep medicine, whereby treatment effects, adverse effects, and net benefits are communicated to medical practitioners in advance, leading them to choose the best possible treatment for each patient. While the advent of such technology-based prediction models and the potential benefits of utilizing the models for patients with OSA and concomitant cardiovascular risks and diseases are acceptable, we must bear in mind that physicians and other medical practitioners are essential players in preventing cardiovascular events in these patients by encouraging them to adhere to a healthy diet, including salt restriction and a reduction in calory intake, physical activity, the maintenance of healthy body weight, smoking cessation, sobriety, and other self-management, all of which are recommended to improve CVH.

### 4.3. Limitations

We acknowledge that this study has several limitations. First, this was a retrospective analysis of a single-center observational study in an urban area with a relatively small sample size. Since sleep disorders can be influenced by occupational and residential settings, we cannot rule out the possibility that a multicenter study that includes rural areas may lead to different results. Second, unknown confounders, such as diet, physical activity, and other lifestyle factors may have affected the results, even after the multivariate analysis. Therefore, our data should be interpreted carefully, and further studies with larger sample sizes are required to confirm our findings. Although the reliability of HOMA-IR depends on the precision of the insulin radioimmunoassay, we lack detailed information on the assay used to measure insulin in this study. Since the majority of the study participants are men, our findings may not be applicable for women. Finally, although this study included subjects without DM or known CVDs, the possibility of asymptomatic or latent CVDs being present cannot be completely excluded.

## 5. Conclusions

Our study demonstrated that the severity of OSA was independently correlated with insulin resistance, as assessed using HOMA-IR in a Japanese population with OSA who do not have DM and CVDs. 

## Figures and Tables

**Table 1:** Baseline characteristics of the study participants.

	*n* = 483
Age, years	55.0 (44, 64)
Men, *n* (%)	439 (90.9)
BMI (kg/m^2^)	26.6 (24.3, 29.4)
Waist circumference (cm)	95.3 ± 12.0
Hypertension, *n* (%)	261 (54.0)
Total cholesterol (mg/dL)	196 (174, 217)
Triglyceride (mg/dL)	139 (98, 197)
High-density lipoprotein cholesterol (mg/dL)	46 (40, 54)
Low-density lipoprotein cholesterol (mg/dL)	115.4 (97.2, 132.6)
Fasting blood glucose (mg/dL)	97 (91, 106)
Glycated hemoglobin (%)	5.4 (5.1, 5.8)
Insulin (μU/mL)	8 (5, 14)
Uric acid (mg/dL)	6.5 (5.5, 7.3)
C-reactive protein (mg/dL)	0.1 (0, 0.2)
HOMA-IR	2.00 (1.25, 3.50)

Continuous data are shown as mean ± standard deviation or median (interquartile range). Categorical data are shown as numbers (%). BMI, body mass index; HOMA-IR, homeostasis model assessment for insulin resistance.

**Table 2:** Polysomnographic findings.

	*n* = 483
AHI (/h)	40.9 (26.5, 59.1)
Awake SO_2_ (%)	96 (94, 96)
Lowest SO_2_ (%)	77 (69, 82)
%TST SO_2_ < 90% (%)	14.6 (3.8, 44.3)
3% ODI (/h)	28.4 (13.3, 50.5)
Arousal index (/h)	39.1 (26.6, 56.3)
PLM arousal index (/h)	0.3 ± 1.3
Stage 1 (%)	31.4 (23.4, 44.5)
Stage 2 (%)	46.7 ± 12.6
Stage SWS (%)	2.8 (0.8, 6.5)
Stage REM (%)	10.3 (6.9, 14.2)

Data are shown as mean ± standard deviation or median (interquartile range). AHI, apnea-hypopnea index; SO_2_, oxyhemoglobin saturation; TST, total sleep time; ODI, oxygen desaturation index; PLM, periodic eye movement; SWS, slow-wave sleep; REM, rapid eye movement.

**Table 3:** Relationships between the polysomnographic data and HOMA-IR.

	Coefficient	Standard Error	T	*p*	95% Confidence Interval	Adjusted R-Squared Value	Adjusted R-Squared Value for the Total Model	*p*-Value for the Total Model
AHI	0.0038	0.0016	2.45	0.015	0.0008, 0.0069	0.0675	0.313	<0.0001
3% ODI,	0.0030	0.0013	2.27	0.024	0.0004, 0.0057	0.0742	0.309	<0.0001
lowest SO_2_,	−0.0062	0.0022	−2.88	0.004	−0.010, −0.0020	0.0612	0.312	<0.0001
%TST SO_2_ < 90%,	0.0036	0.0010	3.46	0.001	0.0016, 0.0056	0.1092	0.318	<0.0001
arousal index	0.0034	0.0016	2.20	0.028	0.0004, 0.0065	0.0473	0.308	<0.0001

Age, sex, and BMI were included in each model. AHI, apnea-hypopnea index; ODI, oxygen desaturation index; SO_2_, oxyhemoglobin saturation; TST, total sleep time; BMI, body mass index.

**Table 4:** Results of multiple regression analyses for the relationships between each index of OSA severity and HOMA-IR.

	Coefficient	Standard Error	T	*p*	95% Confidence Interval	Adjusted R-Squared Value	Adjusted R-Squared Value for the Total Model	*p*-Value for the Total Model
AHI	0.0015	0.000608	2.48	0.014	0.0003, 0.0027	0.0742	0.319	<0.0001
3% ODI,	0.0011	0.000566	1.91	0.057	−0.000037, 0.002189	0.0798	0.315	<0.0001
lowest SO_2_,	−0.0026	0.000994	−2.61	0.009	−0.004550, −0.000643	0.0660	0.320	<0.0001
%TST SO_2_ < 90%,	0.0014	0.000465	2.99	0.003	0.000475, 0.002304	0.1148	0.323	<0.0001
arousal index	0.0011	0.000632	1.79	0.074	−0.000110, 0.002375	0.0487	0.313	<0.0001

Age, sex, BMI, waist circumference, and presence of hypertension were included in each model. AHI, apnea-hypopnea index; ODI, oxygen desaturation index; SO_2_, oxyhemoglobin saturation; TST, total sleep time; BMI, body mass index.

## Data Availability

The deidentified participant data will be shared by the corresponding author upon reasonable request.
